# Serum Levels of Oxylipins in Achilles Tendinopathy: An Exploratory Study

**DOI:** 10.1371/journal.pone.0123114

**Published:** 2015-04-13

**Authors:** Sandra Gouveia-Figueira, Malin L. Nording, Jamie E. Gaida, Sture Forsgren, Håkan Alfredson, Christopher J. Fowler

**Affiliations:** 1 Department of Pharmacology and Clinical Neuroscience, Pharmacology Unit, Umeå University, Umeå, Sweden; 2 Department of Chemistry, Umeå University, Umeå, Sweden; 3 Discipline of Physiotherapy, University of Canberra, Bruce, ACT, Australia; 4 Department of Physiotherapy, Monash University, Melbourne, VIC, Australia; 5 Department of Integrative Medical Biology, Section for Anatomy, Umeå University, Umeå, Sweden; 6 Department of Surgical and Perioperative Sciences, Sports Medicine Unit, Umeå University, Umeå, Sweden; University of Sevilla, SPAIN

## Abstract

**Background:**

Linoleic acid-derived oxidation products are found in experimental pain models. However, little is known about the levels of such oxylipins in human pain. In consequence, in the present study, we have undertaken a lipidomic profiling of oxylipins in blood serum from patients with Achilles tendinopathy and controls.

**Methodology/Principal findings:**

A total of 34 oxylipins were analysed in the serum samples. At a significance level of P<0.00147 (<0.05/34), two linoleic acid-derived oxylipins, 13-hydroxy-10E,12Z-octadecadienoic (13-HODE) and 12(13)-dihydroxy-9Z-octadecenoic acid (12,13-DiHOME) were present at significantly higher levels in the Achilles tendinopathy samples. This difference remained significant when the dataset was controlled for age, gender and body-mass index. In contrast, 0/21 of the arachidonic acid- and 0/4 of the dihomo-γ-linolenic acid, eicosapentaenoic acid or docosahenaenoic acid-derived oxylipins were higher in the patient samples at this level of significance. The area under the Receiver-Operator Characteristic (ROC) curve for 12,13-DiHOME was 0.91 (P<0.0001). Levels of four N-acylethanolamines were also analysed and found not to be significantly different between the controls and the patients at the level of P<0.0125 (<0.05/4).

**Conclusions/Significance:**

It is concluded from this exploratory study that abnormal levels of linoleic acid-derived oxylipins are seen in blood serum from patients with Achilles tendinopathy. Given the ability of two of these, 9- and 13-HODE to activate transient receptor potential vanilloid 1, it is possible that these changes may contribute to the symptoms seen in Achilles tendinopathy.

## Introduction

The oxylipins are a family of oxidised lipids derived from polyunsaturated fatty acids that have a wide range of biological activities. Most studied are the cyclooxygenase- and lipoxygenase-derived arachidonic acid metabolites, but linoleic acid metabolites have also been shown to have important biological effects including the activation of transient potential receptor vanilloid 1 (TRPV1) receptors involved in pain transmission [1,2]. Levels of linoleic acid-derived oxylipins are increased in several experimental pain conditions [2–5], but to our knowledge only two studies have investigated levels of linoleic acid derivatives in human pain. Stevens *et al*. [6] found increased serum levels of 9- and 13-HODE and 9- and 13-oxo-ODE (for explanation of abbreviations, see S1 Table), expressed as ratios to linoleic acid, in a small study of patients with chronic pancreatitis *vs*. symptomatic controls (patients with abdominal pain but not chronic pancreatitis). The levels were also correlated with disease severity as determined by endoscopic ultrasound [6]. Levels of 9- and 13-HODE, but not 9- and 13-oxo-ODE, were also higher in the pancreatic fluid of nine patients with mild chronic pancreatitis compared with nine symptomatic controls [6]. In a study investigating dental pulp biopsy samples from patients with inflammatory dental pain, Ruparel *et al*. [7] did not measure endogenous levels of linoleic acid-derived oxylipins, but instead incubated the tissue with [^14^C]linoleic acid and quantified the oxylipins produced. They reported a ~60% increase in the production of [^14^C]oxylipins compared to control. This increase could be prevented by the CYP / lipoxygenase inhibitor ketoconazole.

Achilles tendinopathy is a painful condition usually resulting from chronic overuse and associated with an increase in the number of tenocytes, morphological changes in their appearance, neovascularisation and eventually signs of degeneration, whereas overt signs of chronic inflammation are lacking [8]. Within the tendon, changes in levels of cytokines such as transforming growth factor β and signalling molecules such as substance P are seen [9]. With respect to oxylipins, a microdialysis study reported that levels of prostaglandin E_2_ in tendons from patients with tendinopathy were not significantly different to those for healthy controls, whereas levels of glutamate, an algogenic agent, were higher [10].

The studies described above investigated abnormalities within the tendon tissue. However, changes in the blood have also been reported. In a study on 22 cases of Achilles tendinopathy, the blood serum levels of soluble tumour necrosis factor receptor I (sTNFRI) were significantly correlated with the level of total physical activity, whereas this was not the case for the 19 control cases investigated [11]. Given the paucity of information concerning the levels of linoleic acid-derived oxylipins in painful conditions in general, and in Achilles tendinopathy in particular, we took the opportunity to investigate oxylipin levels in previously unused serum samples from the study of [11]. The results of this exploratory study indicate that there is a pattern of increased levels of linoleic acid-derived, but not arachidonic acid-derived oxylipins in the serum samples from the Achilles tendinopathy patients compared to the controls.

## Methods

### Sample collection

The samples used were originally collected for a previous study [11]. In brief, blood samples were collected from patients with chronic tendon pain who had not responded to conservative treatment and whose diagnosis as Achilles tendinopathy was made by an experienced orthopaedic surgeon (H.A.). Diagnosis was confirmed by ultrasound imaging using an Acuson Sequoia 512 imager (Siemens AG, Munich, Germany). The characteristic changes seen in the patients, but not the controls, included a widening of the anterior-posterior diameter of the Achilles midportion accompanied by structural abnormalities of the anterior tendon margin. Nine of the patients had unilateral tendon abnormalities (2 left, 7 right), whilst the remaining six patients had bilateral symptoms. Three of the patients with Achilles tendinopathy were receiving medication with statins. Controls were defined as healthy individuals with normal ultrasound tendon findings and no history of tendon pain. None of the participants were smokers. The blood samples were collected by antecubital venopuncture between 07:00 and 09:00 hours following a 10–12 h overnight fast. The samples were collected into a 4 mL serum separating tube (Vacuette 454067, Greiner Bio-One GmbH, Kremsmünster, Austria), and after 30 min at RT, the tube was centrifuged at 1300 g x 10 min. Serum was then stored in aliquots at -80°C. Participants gave verbal consent to participate in the research after reading an explanatory statement and receiving a verbal summary of the project. The study, including the method of consent, was approved by the Ethical Committee at the Faculty of Medicine, Umeå University, and by the Regional Ethical Review Board in Umeå. The protocol used conformed to the principles expressed in the Declaration of Helsinki. In the original study [11], the number of Achilles tendinopathy patients and controls was 22 and 19, respectively. Aliquots from 16 controls and 15 Achilles tendinopathy patients were available for the present study. The serum lipid profiles (cholesterol, triglycerides (TG), high- and low density lipoproteins (HDL and LDL)) used here have been reported previously as part of a larger study [12]. Plasma levels of brain-derived neurotrophic factor (BDNF) and sTNFRI levels in the database were taken from [11]. The demographics of the cases used in the present study are summarised in Table 1.

**Table 1:** Description of the study population.

	Control	Achilles tendinopathy	P value
**Sex (♀, ♂)**	5, 11	6, 9	0.72^##^
**Age (years)**	47 (24–72)	48 (24–60)	0.94
**Height (cm)**	177 (166–190)	175 (158–189)	0.41
**Weight (kg)**	78 (58–112)	84 (66–108)	0.24
**BMI (kg/m** ^2^ **)**	24.8 (20.0–31.1)	27.4 (23.4–32.9)	**0.032**
**Waist (cm)**	89 (75–112)	96 (84–118)	0.075
**Hip (cm)**	100 (92–108)	105 (94–119)	**0.033**
**tMets (meh/w)** ^#^	152 (84–229)	141 (99–238)	0.97
**Cholesterol (mmol/L)**	5.2 (3.8–8.0)	5.0 (3.4–6.1)	0.57
**TG (mmol/L)** ^#^	0.76 (0.45–5.8)	0.92 (0.37–3.0)	0.42
**HDL (mmol/L)** ^#^	1.3 (1.1–3.0)	1.5 (0.8–2.0)	0.99
**LDL (mmol/L)**	3.1 (1.9–4.8)	3.1 (1.4–4.2)	0.95
**BDNF (ng/ml)** ^#^	14 (0.83–20)	16 (2.1–23)	0.35
**sTNFR1 (ng/ml)** ^#^	1.1 (0.077–1.7)	1.1 (0.86–1.6)	0.76

Abbreviation: tMET, physical activity, estimated in metabolic equivalent hours per week. Unless otherwise indicated, values are given as means with ranges in brackets, and statistical significance was assessed using a two-tailed t-test.

^#^One or both of the groups did not pass the D’Agostino & Pearson omnibus normality test, and so the values are given as medians and ranges, with statistical significance being assessed using a two-tailed Mann-Whitney U test.

^##^Fishers exact test. Control, N = 16 except for tMet (N = 11) and LDL (N = 15). Achilles tendinopathy, N = 15 except for waist, hip and tMet (N = 14), cholesterol, TG, HDL and LDL (N = 13).

### Analysis of oxylipins

The oxylipins were analysed by ultra performance liquid chromatography (UPLC) coupled to tandem mass spectrometry (MS/MS) according to previously published protocols [13]. Briefly, the blood serum samples were subjected to solid phase extraction (SPE) using deuterated internal standards to mimic the endogenous oxylipins throughout the sample preparation procedure. Furthermore, by using deuterated internal standards, quantification of each oxylipin species using the stable isotope dilution method was facilitated. To extract the oxylipins, the blood serum samples (120 μL) were loaded onto SPE Waters Oasis HLB cartridges (60 mg sorbent, 30 μm particle size), which were eluted with 2 mL methanol and 2 mL ethyl acetate into polypropylene tubes containing 6 μL of a glycerol solution (30% in methanol). Glycerol was used as a trap solution for the oxylipins. The SPE eluates were evaporated under vacuum (MiniVac system, Farmingdale, NY, USA) and residues were then reconstituted in 110 μL methanol containing a recovery standard. UPLC-MS/MS analysis was performed immediately using a Waters BEH C18 column (2.1 mm x 150 mm, 2.5 μm particle size) and the mass analysis was done on an Agilent 6490 Triple Quadrupole system equipped with the iFunnel Technology source (Agilent Technologies, Santa Clara, CA, USA) in negative multiple reaction monitoring (MRM) mode. The MassHunter Workstation software was used manually to integrate each peak used for quantification. In the original protocol, Yang et al. [13] reported have in detail reported the limits of quantification, intra- and inter-day accuracy and precision. Our ranges were similar to theirs: limit of quantification: 0.0005–4.2 pg on column, inter- and intra-day accuracy: 85–115% and inter- and intraday precision: 0.1–17% for all but five compounds. For the five compounds in question (9,10,13-TriHOME, PGF_2α_, 12(13)-EpOME, 5(6)-EET and 12-oxo-ETE), the accuracy ranged from 48 to 175% at the lowest quality control level (30 pg). Since, with the exception of 12(13)-EpOME, the recovered pg on the columns was around or below this value, the results for these compounds should be considered as being less robust than for the other oxylipins.

For samples where the recovered peak was below the limit of detection, a value of zero was given. Since we use non-parametric statistics, the absolute values of these samples are not required, since they are ranked below those samples whose values are above the limit of detection.

### Analysis of *N*-acylethanolamines

The *N*-acylethanolamines were analysed according to Gouveia-Figueira and Nording [14]. In short, the blood serum samples (300 μL) were extracted using the oxylipin SPE protocol described above, but with *N*-acylethanolamine specific deuterated internal standards. The SPE elutions were evaporated and reconstituted in 110 μL of methanol containing a recovery standard. UPLC-MS/MS analysis was performed immediately using a 2.1 mm× 150 mm Waters BEH C18 column with a 2.5 μm particle size and a Waters triple quadrupole MS (Micromass Quattro Ultima) operating in positive MRM mode was used for MS analysis. For UPLC gradient and MS instrument specific parameters, MRM transitions etc, see [14]. The stable isotope dilution method was used to quantify the peaks by the MassLynx software. For details concerning the limits of quantification, intra- and inter-day accuracy and precision, see [14].

### Statistics

Basal statistical measures (D'Agostino and Pearson omnibus normality tests, Mann-Whitney U-tests, Spearman’s regression) and Receiver-Operator Characteristic (ROC) curves were undertaken using the statistical package built into the GraphPad Prism computer programme for the Macintosh (v6, GraphPad Software Inc., San Diego, CA, USA). Principal components analysis and univariate general linear model analyses were undertaken using the IBS SPSS Statistics package, version 22. For the principal components analysis, the data was mean-centered and Pareto-scaled (for details of different methods of data treatment in metabolomics analyses, see [15]).

## Results

### Patient characteristics

The characteristics of the patients are summarised in Table 1. There were no significant differences for the populations sampled here with respect to age, height, weight, waist, physical activity (tMet, estimated in metabolic equivalent hours per week), cholesterol, TG, HDL, LDL, BDNF or sTNFR1 levels. However, the Achilles tendinopathy patients had a significantly higher BMI and hip size than the controls (Table 1). The Achilles tendinopathy patients reported a median pain duration of 24 months (range 6–120 months, N = 14 since one value was not available in the database), whereas the controls did not report tendon pain.

### Characterisation of the serum oxylipin profile of the sample

A total of 37 oxlipins were investigated in the serum samples. The median and range of values for all 37 oxylipins are given in S1 Table, together with their full chemical names and comparative values from two recent publications from other groups. Of these, three were not used: 17(R)-HDoHE, 9-HETE and 5-oxo-ETE, since too many cases had values below the limit of detection, (30, 19 and 20 of 31 cases, respectively). For the remaining 34 parameters, all but seven (PGF_2α_, 8,9-DHET, 14,15-DHET, 5,6-EET, 8,9-EET, 11,12-EET and Resolvin D2) failed to pass the D'Agostino and Pearson omnibus normality test, and so the data has been presented as medians and ranges in S1 Table together with examples from the literature.

For the whole dataset, bivariate non-parametric correlations were undertaken between the sample characteristics (age, tMets, BMI, cholesterol, TC, HDL, LDL, BDNF and sTNFRI) and the plasma oxylipin levels (Table 2). For the linoleic acid (18:2) derivatives, only 3 of 81 possible correlations were significant at P<0.05. However, 7/22 and 8/22 of the arachidonate derivatives showed significant positive correlations with BMI and TG levels, respectively (Table 2). With few exceptions, no significant associations of the oxylipins with age, tMets, HDL, LDL, BDNF or sTNFR1 were seen (Table 2). Note that the correlations implicitly assume good individual test-retest reliability of all the measures in this patient population.

**Table 2:** Non-parametric bivariate correlation coefficients between serum oxylipin or *N*-acylethanolamine levels and the age, physical activity, pain duration (Achilles tendinopathy patients), BMI, blood lipid status, BDNF and sTNFR1 levels in the dataset .

	Age (n = 31)	tMets (n = 25)	PaD-A (n = 14)	BMI (n = 31)	Chol (n = 29)	TG (n = 29)	HDL (n = 29)	LDL (n = 28)	BDNF (n = 31)	sTNFRI (n = 31)
**Oxylipin:**										
***Linoleic acid (18*:*2) derivatives***										
**9(S)-HODE**	-0.09	-0.16	0.32	0.01	-0.12	-0.03	0.15	-0.12	0.23	-0.02
**13-HODE**	-0.01	-0.19	0.35	0.03	-0.12	0.00	0.26	-0.09	0.25	0.00
**9,10-DiHOME**	-0.02	0.01	0.29	-0.15	-0.13	-0.15	0.45*	-0.16	0.27	0.18
**12,13-DiHOME**	0.09	-0.04	0.34	0.08	-0.11	-0.07	0.23	-0.12	0.29	0.05
**9,10,13-TriHOME**	-0.06	-0.34	-0.20	0.02	0.01	0.01	0.01	0.03	0.17	-0.06
**9,12,13-TriHOME**	-0.03	-0.27	-0.04	-0.03	-0.07	-0.06	0.23	-0.14	0.16	-0.07
**9,10-EpOME**	-0.02	0.27	0.10	0.43*	0.02	0.26	-0.26	0.16	0.11	0.23
**12,13-EpOME**	0.05	0.21	0.32	0.37*	-0.03	0.15	-0.15	0.10	0.10	0.16
**13-oxo-ODE**	-0.08	-0.08	0.25	0.21	-0.19	-0.11	0.10	-0.08	0.15	0.00
***Dihomo-γ-linolenic acid (20*:*3) derivative***										
**15(S)-HETrE**	0.10	-0.22	0.22	0.40*	0.26	0.33	-0.35	0.28	0.28	0.05
***Arachidonic acid (20*:*4) derivatives***										
**PGD**_2_	-0.12	0.26	0.48	0.18	0.18	0.46*	-0.35	0.12	-0.01	0.04
**PGE**_2_	0.16	0.30	0.36	0.31	0.31	0.40*	-0.31	0.27	0.14	0.06
**PGF**_2α_	0.31	-0.14	-0.17	0.01	0.16	-0.26	0.24	0.13	0.06	0.01
**TXB**_2_	0.03	0.28	0.43	0.30	0.26	0.50**	-0.38*	0.24	0.21	0.11
**LTB**_4_	0.03	0.23	0.06	-0.07	0.19	0.23	0.03	0.20	0.15	0.21
**5-HETE**	0.16	0.02	0.14	0.32	0.00	0.26	-0.04	0.02	0.24	0.10
**8-HETE**	0.11	0.17	0.39	0.44*	0.27	0.30	-0.15	0.14	-0.06	0.01
**11-HETE**	0.02	0.14	0.45	0.44*	0.28	0.54**	-0.45*	0.30	0.24	0.16
**12-HETE**	0.09	0.45*	0.39	0.40*	0.23	0.51**	-0.25	0.11	0.21	0.28
**15-HETE**	0.03	0.23	0.45	0.46*	0.17	0.49**	-0.36	0.15	0.19	0.12
**20-HETE**	0.30	0.14	0.07	0.33	0.27	0.35	0.05	0.14	0.06	-0.05
**12-oxo-ETE**	0.21	0.23	0.30	0.22	0.28	0.36	0.13	0.12	0.08	0.04
**15-oxo-ETE**	0.10	-0.21	-0.09	0.04	-0.12	0.14	0.27	-0.26	0.38*	-0.21
**5,6-DHET**	0.24	0.18	0.02	0.25	0.24	0.48**	-0.11	0.01	0.34	0.30
**8,9-DHET**	0.21	0.26	-0.04	0.33	0.36	0.28	-0.11	0.35	0.31	0.03
**11,12-DHET**	0.26	0.13	0.21	0.43*	0.05	0.17	-0.04	0.13	0.26	-0.03
**14,15-DHET**	0.25	0.13	0.02	0.61***	0.20	0.45*	-0.34	0.19	0.39*	0.02
**5(6)-EET**	-0.02	0.32	0.12	0.29	-0.05	0.32	0.01	-0.13	0.25	0.22
**8(9)-EET**	-0.03	0.25	0.02	0.18	-0.05	0.20	0.07	-0.17	0.22	0.13
**11(12)-EET**	0.06	0.29	0.08	0.29	-0.03	0.29	0.05	-0.09	0.26	0.28
**14(15)-EET**	-0.05	0.12	0.16	0.12	-0.32	0.11	0.25	-0.40*	0.17	0.06
***Eicosapentaenoic acid (20*:*5) derivative***										
**12(S)-HEPE**	0.36*	0.31	0.38	0.38*	0.50**	0.42*	-0.15	0.33	0.15	0.22
***Docosahexaenoic acid (22*:*6) derivatives***										
**Resolvin D1**	-0.05	0.01	0.11	-0.01	-0.27	-0.28	0.11	-0.16	0.00	0.09
**Resolvin D2**	-0.14	0.02	0.03	0.17	-0.03	0.13	-0.28	-0.13	0.15	0.17
***N-acylethanoalamines***										
**PEA**	0.00	0.06	-0.05	0.01	-0.14	-0.25	0.23	-0.10	-0.08	-0.23
**OEA**	0.03	-0.05	0.16	0.35	-0.11	0.21	-0.08	-0.08	0.00	0.19
**LEA**	-0.07	0.14	-0.03	0.10	-0.06	-0.06	-0.12	-0.03	0.16	-0.07
**SEA**	0.13	-0.02	0.27	0.19	-0.17	0.07	-0.12	-0.16	0.10	-0.04

Abbreviations: tMets, physical activity, estimated in metabolic equivalent hours per week; PaD-A, pain duration for the patients with Achilles tendinopathy; BMI, body mass index; Chol, serum cholesterol; TG, serum triglycerides; HDL and LDL, serum high and low density lipoprotein; BDNF and sTNFRI, serum levels of brain-derived neurotrophic factor (BDNF) and soluble tumour necrosis factor receptor I, respectively. Significance levels are: *P<0.05, **P<0.01, ***P<0.001. For OEA, the sample sizes, with the exception of PaD-A are one smaller than those shown at the top of the Table due to one missing value.

### Differences in oxylipin levels between controls and patients with Achilles tendinopathy

**Fig 1:**
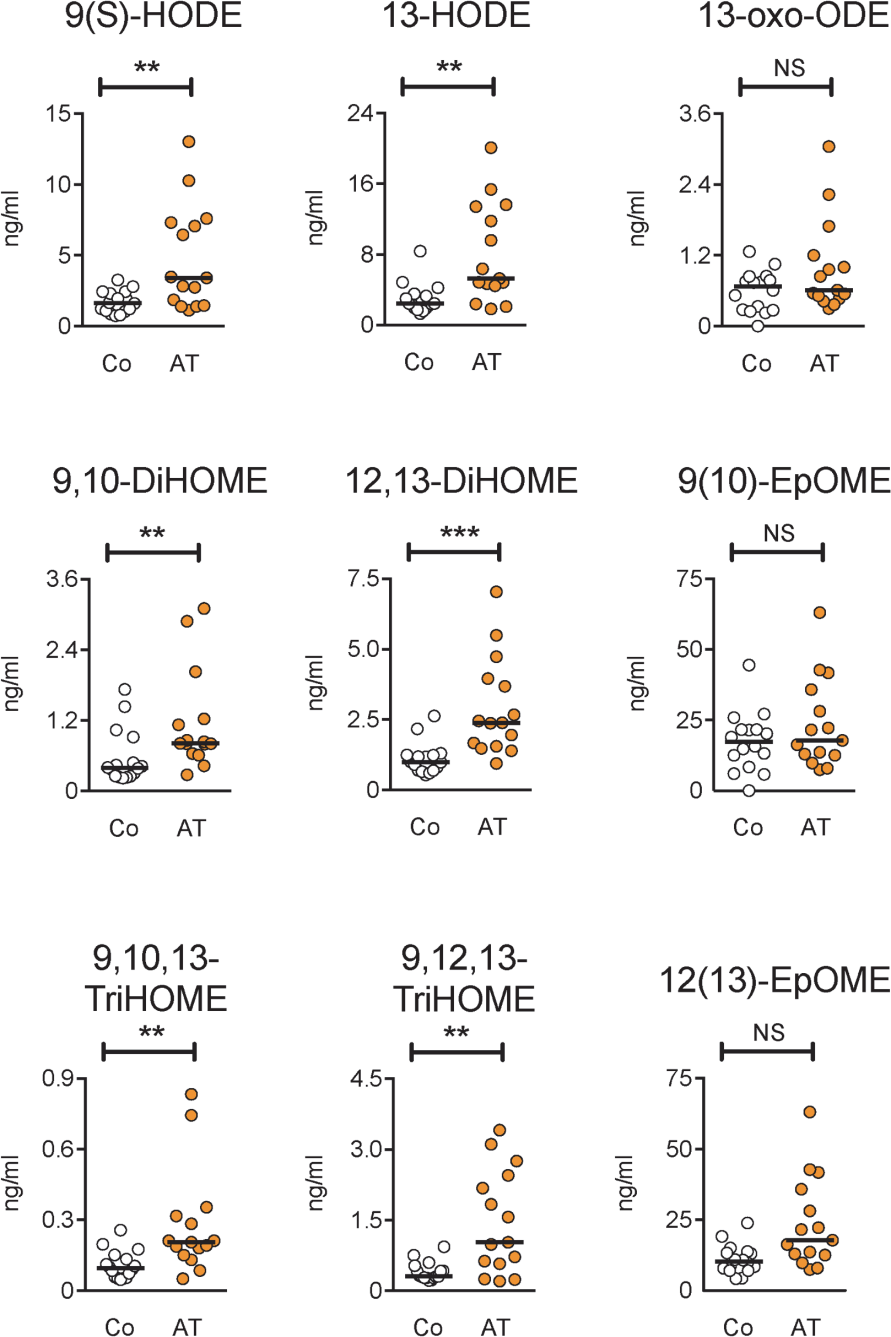
Scatterplots for the oxylipins derived from linoleic acid. Co, controls; AT, Achilles tendinopathy. Significance levels are from the Mann-Whitney U-test summarised in Table 1: ***P<0.001, **P<0.01, ^NS^P>0.05.

**Fig 2:**
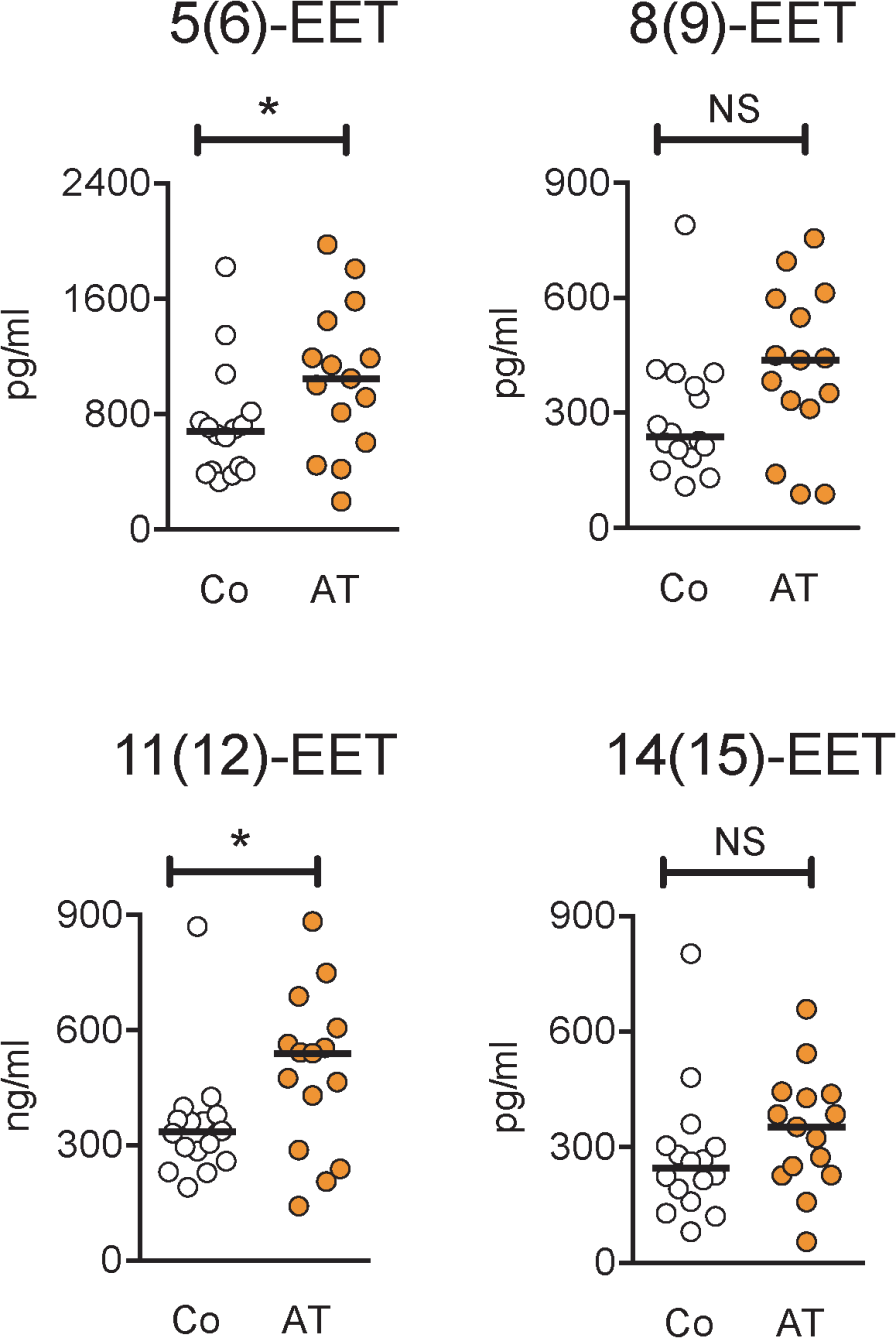
Scatterplots for the epoxy-5Z,8Z,14Z- eicosatrienoic acid oxylipins. Co, controls; AT, Achilles tendinopathy. Significance levels are from the Mann-Whitney U-test summarised in Table 1: *P<0.05, ^NS^P>0.05.

Differences in oxylipin levels were assessed using a non-parametric Mann-Whitney U-test (Table 3). Of the 34 oxylipins investigated, significant differences were seen for eight: six belonging to the linoleic class of compounds (9,10,13- and 9,12,13-TriHOME, 9,10- and 12,13-DiHOME, 9(S)- and 13-HODE, all P<0.01; see Fig 1 for scatterplots of the data) and two arachidonic acid derivatives (5,6- and 11,12-EET, both P<0.05; Fig 2). It should be noted that these significance values have not been adjusted for multiple comparisons. Within the linoleic acid derivatives, there are 9 comparisons. Using the conservative Bonferroni correction, a threshold significance level of 0.0056 would be appropriate, and five of the oxylipins reach this significance. In contrast, for the 21 arachidonic acid derivatives, none of the lipids are even close to the threshold significance level of 0.0024. At an even more conservative level (which would raise the risk of Type II errors), a significance level of 0.00147 (Bonferroni correction for 34 comparisons, i.e. all oxylipins irrespective of class) would still show significant effects of 13-HODE and 12,13-DiHOME, with 9,10,13-TriHOME right on the boundary (Table 3). For the Achilles tendinopathy patients, there were no significant correlations between the duration of their tendon pain (median 24 months, range 6–12 months) and any of the oxylipins (Table 2), although it should be noted as a caveat that the sample size was small.

**Table 3:** Oxylipin levels (pg/mL) in the blood serum samples from controls and patients with Achilles tendinopathy.

	**Controls**			**Achilles Tendinopathy**			
**Oxylipin**	**Median**	**IQR**	**Range**	**Median**	**IQR**	**Range**	**P value**
*Linoleic acid (18*:*2) derivatives*							
**9(S)-HODE**	1640	1350	744–3270	3410	5850	1130–13000	**0.0036**
**13-HODE**	2410	1580	1290–8370	5270	8970	1820–20100	**0.0012**
**9,10-DiHOME**	394	553	221–1730	813	588	277–3100	**0.0072**
**12,13-DiHOME**	995	551	536–2630	2390	2410	946–7040	**<0.0001**
**9,10,13-TriHOME**	96	92	47–257	206	166	50–833	**0.0015**
**9,12,13-TriHOME**	314	229	218–932	1030	1880	203–3410	**0.0055**
9(10)-EpOME	17400	12200	0–44500	17800	23300	7430–63100	**0.40**
12(13)-EpOME	10300	6400	4230–23900	14200	12500	4400–35200	0.085
13-oxo-ODE	673	554	0–1270	609	733	299–3040	0.17
*Dihomo-γ-linolenic acid (20*:*3) derivative*							
15(S)-HETrE	42	44	0–120	36	84	0–316	0.68
*Arachidonic acid (20*:*4) derivatives*							
PGD_2_	57	84	14–211	79	136	0–427	0.95
PGE_2_	141	136	43–462	119	339	14–1760	0.67
PGF_2α_	2530	2240	231–6090	1960	1240	140–5210	0.85
TXB_2_	7780	15500	108–37100	5640	25700	0–135000	0.85
LTB_4_	43	58	8–213	62	113	9–262	0.10
5-HETE	261	167	158–569	468	562	117–1410	0.085
8-HETE	7060	5010	0–21200	8350	20400	0–52400	0.095
11-HETE	179	219	36–1050	200	821	17–2560	0.88
12-HETE	8080	7900	2610–38600	11800	45100	58–152000	0.57
15-HETE	520	458	182–1800	490	1760	156–4320	0.65
20-HETE	831	648	0–1900	1370	1900	55–3530	0.30
12-oxo-ETE	350	210	0–711	409	351	0–1270	0.63
15-oxo-ETE	62	41	18–110	74	89	0–277	0.54
5,6-DHET	132	59	77–390	109	78	41–217	0.49
8,9,DHET	55	19	33–148	80	44	37–149	0.071
11,12-DHET	104	57	65–193	118	67	47–257	0.57
14,15-DHET	399	111	160–555	427	297	222–699	0.47
**5(6)-EET**	679	394	333–1820	1050	844	197–1980	**0.045**
8(9)-EET	237	206	109–791	438	288	89–755	0.078
**11(12)-EET**	335	111	191–870	540	318	142–884	**0.030**
14(15)-EET	245	136	81–803	352	210	54–658	0.093
*Eicosapentaenoic acid (20*:*5) derivative*							
12(S)-HEPE	642	510	112–1050	692	1350	60–5600	0.33
*Docosahexaenoic acid (22*:*6) derivatives*							
Resolvin D1	0	23	0–51	27	40	0–104	0.15
Resolvin D2	212	337	0–470	131	225	0–358	0.18

Shown are medians, interquartile range and total ranges for the lipids (N = 16 for controls, N = 15 for the Achilles tendinosis group), classified according to the polyunsaturated fatty acid from which they are derived. P values are derived from the Mann-Whitney U-test.

**Fig 3:**
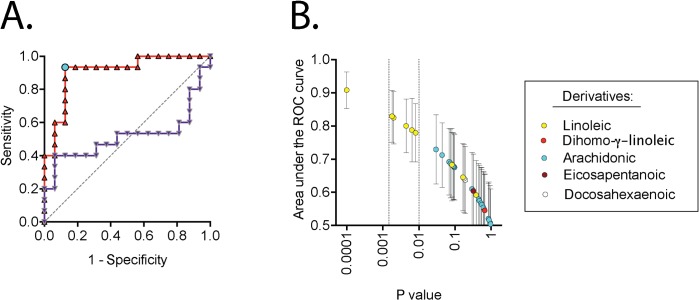
Receiver operated curves (ROC) for the oxylipins. In Panel A, two individual examples are shown for 12,13-DiHOME (red line) and 11-HETE (purple line). The area under the ROC curves for these lipids were 0.91 (95% CI 0.80–1.02, P = 0.0001) and 0.52 (95% CI 0.29–0.74, P = 0.87), respectively. The dotted line is for an area under the ROC curve of 0.5, i.e. no predictive value. The turquoise circle in the ROC curve for 12,13-DiHOME shows the optimum cut-off value (the Youden score). In Panel B, the area under the ROC curves (means ± SE) are plotted against the P values from the ROC analyses for all the oxylipins, colour-coded upon the basis of the fatty acid group to which they belong. The vertical lines delineate P values of 0.00147 (= 0.05/34) and 0.01. The three oxylipins with the highest P value and area under the ROC curves are (left to right) 12,13-DiHOME, 13-HODE and 9,10,13-TriHOME.

In their article discussing how to avoid data misinterpretation in metabolomics studies, Broadhurst and Kell [16] recommend the use of Receiver-Operator Characteristic (ROC) curves. ROC curves are non-parametric in nature and were originally developed to aid interpretation of radar signals. They consider specificity (true negatives) and sensitivity (true positives) identified at a given cut-off value (i.e. for cases with a score equal to, or higher than, the selected value) of the parameter in question. The area under the ROC curve, plotted as sensitivity *vs*. 1-specificity, can then be calculated. For a parameter with absolutely no diagnostic value, a straight line with an area of 0.5 will be found. The maximum value is 1.0, so the nearer this value, the better the diagnostic usefulness of the parameter in question, with value ranges of >0.9, 0.7–0.9, 0.5–0.7 and 0.5 being considered high, moderate, low accuracy and a chance result, respectively [17]. In line with the recommendation of [16], we determined the area under the ROC curves for all 34 oxylipins. The data are summarised for all the oxylipins in Fig 3, with examples from two oxylipins (one with the best outcome, and one with a value not significantly different from a chance result) being shown in panel A for illustrative purposes. Unsurprisingly, the ROC curves mirrored the Mann-Whitney U-tests shown in Table 1. However, 12,13-DiHOME stood out with an excellent area under the ROC curve (0.91, 95% CI 0.80–1.02, P = 0.0001). From the data shown in Fig 3A, an optimal cut-off value can be determined as the maximum value of specificity + sensitivity -1 (the Youden score, in this case >1353 pg/ml for 12,13-DiHOME). This is shown as the turquoise circle in Fig 3A. Fourteen of the fifteen cases with Achilles tendinopathy had plasma levels of 12,13-DiHOME higher than this cutoff value, whereas only two of the sixteen controls exceeded this value (P<0.0001, Fisher’s exact test).

### Do gender or BMI influence the different HODE, DiHOME or TriHOME levels in controls vs. Achilles tendinopathy patients?

Given that the samples were from both females and males, and that the BMI of the total sample was different for controls and Achilles tendinopathy patients (Table 1), the influence of these variables upon the HODE, DiHOME or TriHOME derivatives was assessed. Non-parametric analyses taking into account both gender and BMI, the latter as a continuous variable, are beyond the capabilities of the current authors. However, the log_10_ values of HODE, DiHOME or TriHOME derivatives passed the D'Agostino and Pearson omnibus normality test (there were no values under the detection limit, which would otherwise have been an issue). In consequence, general linear models with the log_10_ oxylipin as dependent variable, diagnosis and gender as fixed factors and with age and BMI as covariates were investigated. Additionally, we reduced all six derivatives to a single variable (“PCA factor”, scatterplot and ROC plot of this variable are shown in S1 Fig) using a principal component analysis of the mean-centered and Pareto-scaled log_10_ values. Hip size was not used as a covariate since its high level of correlation with BMI in the sample (Pearson r = 0.75, P<0.0001, N = 30) raises issues of multicollinearity. In all cases, the general linear models returned significant effects of the diagnosis, but not gender (with the exception of log_10_ 9,10,13-TriHOME as dependent variable), diagnosis x gender, age or BMI, on the oxylipin derivatives or the PCA factor derived from them (Table 4).

**Table 4:** Univariate general linear model significance levels for the log_10_ values of HODE, DiHOME, TriHOME derivatives and for the derived PCA factor.

**Oxylipin**	**BMI**	**Age**	Diagnosis	**Gender**	Diagnosis * Gender
log_10_ 9(S)-HODE	0.807	0.471	**0.0059**	0.172	0.481
log_10_ 13-HODE	0.923	0.524	**0.0065**	0.069	0.783
log_10_ 9,10-DiHOME	0.300	0.954	**0.012**	0.199	0.683
log_10_ 12,13-DiHOME	0.273	0.543	**0.00017**	0.816	0.740
log_10_ 9,10,13-TriHOME	0.420	0.807	**0.0013**	**0.038**	0.214
log_10_ 9,12,13-TriHOME	0.216	0.910	**0.00046**	0.101	0.147
PCA factor	0.361	0.834	**0.00022**	0.091	0.531

The PCA factor was determined from the mean-centered and Pareto-scaled log_10_ oxylipin values for the six derivatives. A single factor with eigenvalue >1 was returned, with a component matrix log_10_ 9(S)-HODE, 0.924; log_10_ 13-HODE, 0.934; log_10_ 9,10-DiHOME, 0.795; log_10_ 12,13-DiHOME, 0.853; log_10_ 9,10,13-TriHOME, 0.852 and log_10_ 9,12,13-TriHOME, 0.848. The Kaiser-Meyer-Olkin Measure of sampling adequacy was 0.818. The P values for the corrected model and intercepts are not shown but were significant in all cases except for the intercept for the PCA factor (which is mean-centered) as dependent variable.

### *N*-acylethanolamine levels in the serum of controls and Achilles tendinopathy patients

**Fig 4:**
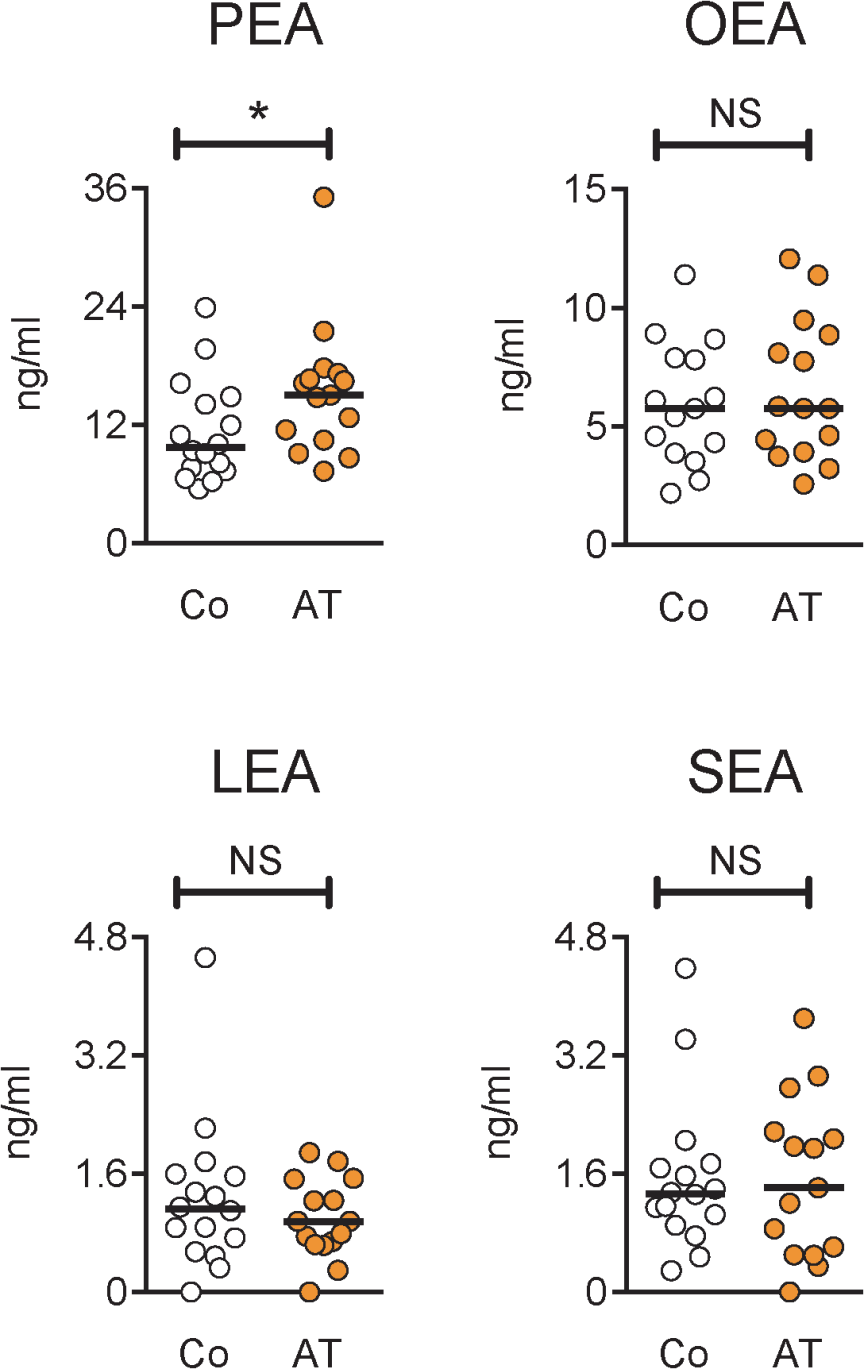
Comparison of *N*-acylethanolamine levels in control and Achilles tendinopathy serum samples. Co, controls; AT, Achilles tendinosis. Significance levels are from the two-tailed Mann-Whitney U-tests, *P = 0.040; ^NS^P>0.5.

It has recently been shown that levels of the *N*-acylethanolamine compounds palmitoylethanolamide (PEA) and stearoylethanolamide (SEA) are higher in interstitial trapezius muscle tissue of patients suffering from myalgia [18]. In order to determine whether levels of these compounds are abnormal in the current samples, we assayed serum samples for *N*-acylethanolamines. There were no significant correlations between their levels and age, BMI, physical exercise, pain duration (Achilles tendinopathy patients), lipid status, BDNF or sTNFRI levels (Table 1). Further, neither SEA, nor the corresponding oleoyl- (OEA) or α-linolenoyl- (LEA) ethanolamides showed significant differences between groups (Fig 4). The median value for PEA was significantly higher in the Achilles tendinopathy group than in the controls, but the level of significance was small (P = 0.040) and did not reach the P<0.0125 level required when a Bonferroni correction was applied. A similar result was seen for the area of the ROC curve (0.72, 95% CL 0.53–0.90, P = 0.040). Indeed, in a two-way robust non-parametric ANOVA with patient category and gender as main effects, the F value for patient category was not significant (P = 0.055) for PEA.

## Discussion

In the present study, the levels of oxylipins and selected *N*-acylethanolamines have been investigated in serum from patients with Achilles tendinopathy and from healthy controls. Reported blood levels of oxylipins vary considerably in the literature, and this is well illustrated by the case of two recent studies [19,20] shown in S1 Table. Our values are in some cases similar to those seen in plasma samples in [19], in others nearer those for plasma samples reported in [20].

The study has one major strength and two important weaknesses that should be clarified at the outset. The strength of the study is that it provides novel information associating a changed pattern of linoleic acid metabolites with a painful disorder where the pain is not accompanied by marked inflammation. The primary weakness of the study is that the samples were originally taken for another purpose [11], rather than being designed for the present purpose. Thus, although they were stored frozen in aliquots to avoid freeze-thaw issues, the limited amounts of sample available meant that we had to prioritise what could be analysed. We chose to look at oxylipins and at selected *N*-acylethanolamines, the latter in view of findings that these are abnormal in human pain [18], at the cost of investigating the levels of the fatty acids from which the oxylipins are derived. Secondly, it should be noted that the patients in the study ranged from 24 to 60 years. Thus, the patients may have different causes for their tendinopathy—some due to athletic overuse, and some who suffer from the tendinopathy despite a more sedentary lifestyle. The study should thus be considered as exploratory, but in this light, it provides important information.

The main finding of the study is that at least two of the linoleic acid derivatives show elevated serum levels in the Achilles tendinopathy cases, whereas none of the derivatives of the other fatty acids show such an elevation. We have stressed the use of the Bonnferoni correction in order not to over-interpret our findings, however it can be argued that this is more appropriate in confirmatory analyses rather than exploratory studies. Certainly, the main effect of the patient category remained significant when the HODE, DiHOME or TriHOME derivatives were packaged into a single variable by use of a principal component analysis.

Two questions arise from these findings: first, why does the Achilles tendinopathy lead to a change in the pattern of linoleic acid-derived oxylipins in the serum; and secondly, do these changes contribute to, or are a result of, the painful symptoms of the disorder? The short answer, of course, is that we do not know, but there are a number of possibilities raised by the current literature. With respect to the former, Achilles tendinopathy in its chronic phase has not been regarded as an inflammatory disorder, although this has been questioned in view of the presence of inflammatory mediators such as interleukins-1 and -6 and transforming growth factor β in this disorder [21,22]. Exercise *per se* also produces an increase in the levels of interleukin 6 and transforming growth factor β_1_ in the blood of healthy subjects [23,24]. Transforming growth factor β can upregulate 5-lipoxygenase activity [25] and so an attractive hypothesis would be where a disrupted cytokine signalling pattern leads to abnormal lipoxygenase activities and thereby a change in the oxylipin concentrations. However, a recent study has reported that a 75-km cycling time trial increased plasma levels of 9 + 13-HODE (and 9,10- + 12,13-DiHOME). The post-exercise HODE levels were not correlated to the post-exercise cytokine concentrations measured in the study (including interleukin-6), but were correlated with the post-exercise levels of F2-isoprostanes, which are markers of exercise-induced oxidative stress [26]. Our data showing a selective effect on the linoleic acid derivatives are also inconsistent with the hypothesis outlined above: an upregulation of 15-lipoxygenase, for example, would be expected to affect 13-HODE levels, but also would be expected to affect levels of other 15-lipoxygenase-derived products, such as 15-HETE (for a schematic of lipid metabolism pathways, see Fig 1 of [27]). Conversely, levels of 9,10- and 12,13-DiHOME, which are derived by CYP450- rather than lipoxygenase- pathways, would not be expected to be changed. Thus, the most likely explanation of the present data is that there is a general mobilisation of linoleoyl-metabolism in Achilles tendinopathy.

Levels of oxylipins can be affected by dietary interventions (see e.g. [20]), and in patients with chronic headache, a dietary reduction in linoleic acid reduces plasma 9- and 13-HODE levels [28]. Adiposity and dyslipidemia are associated with tendinopathy [12,29,30], and our patient sample had a slightly, but significantly, higher BMI than the controls. Dandona et al. [31] reported that 13-HODE levels were approximately threefold higher in nine obese patients (BMI range 32.5–64.4 kg/m^2^) than in 12 normal subjects with a mean BMI of 22.5 kg/m^2^. A 4-week dietary restriction, which produced a mean weight loss of ~4%, reduced levels of this oxylipin and of 9-HODE by about 50% [31]. Even after dietary restriction, at least 8/9 individuals would still have a BMI >32 (as adjudged from Table 1 of [31]), suggesting that at the lower BMI values in the present study (2/15 had BMI values just above 32), the findings of [31] are not applicable to the present situation. Conversely, Schuchardt et al. [32] reported that levels of 12,13-DiHOME were significantly lower in 20 hyperlipidemic men (mean BMI 27.3 kg/m^2^) compared to 20 normolipidemic men (mean BMI 24.9 kg/m^2^, P = 0.034 vs. the hyperlipidemic men) whereas levels of 9- and 13-HODE and 9,10-DiHOME were not significantly different between the two groups [32]. These authors also found lower levels of 12-HETE in the hyperlipidemic men [32]. Although these changes are in the opposite direction to the present study, they raise the possibility that our findings could be secondary to BMI or lipid profile of the patients, rather than to the tendinopathy itself. However, there was little association between these parameters and the linoleic acid derivatives, and the difference between controls and Achilles tendinopathy patients remained significant even when the data were controlled for BMI (Table 4). In fact, BMI and triglyceride levels were more associated with the arachidonic acid derivatives, rather than the linoleic acid derivatives (Table 2).

With respect to whether or not the changed levels of the linoleic acid derivatives contribute to, or are a result of, the pain experienced by the tendinopathy patients, there is data suggesting that in experimental animals the oxidized metabolites of linoleic acid can activate TRPV1 receptors that are sensitive to capsaicin and that are involved in the gating of painful stimuli (review, see [33]). Most work in this area has been undertaken by the Hargreaves group, who have shown, among other findings, that depolarisation of isolated spinal cords led to a release of 9-HODE which in turn could activate TRPV1 receptors on capsaicin-sensitive trigeminal neurons to give a calcium response [1]. This oxylipin was not able to produce calcium responses in trigeminal neurons from TRPV1-knockout mice [2]. Exposure of skin to noxious heat also results in the production of 9- and 13-HODE [2], and thermal injury to the paw results in increase levels of 9- and 13-HODE, 9- and 13-oxoODE 24 hours later [4]. In the latter case, the use of either antibodies to the HODE derivatives or a TRPV1 receptor antagonist reduced the allodynia produced by the injury. Inflammatory pain produced by complete Freund’s adjuvant injection also involves the oxidized linoleic acid—TRPV1 axis [3]. In contrast, local levels of 9- and 13-HODE are reduced following carrageenan-induced inflammation, although the local administration of the 9- and 13-HODE antibodies did reduce carrageenan-induced hyperalgesia [34]. Less work has been undertaken in humans, but raised plasma levels of 9(10)-EpOME have been reported in patients with severe burns [35], and inflamed dental pulp more efficiently converts [^14^C]linoleic acid to its oxidised metabolites than normal dental pulp [7]. This increase was blocked by ketoconazole [7], suggesting involvement of CYP-pathways, although this compound can also block 5-lipoxygenase [36]. These studies did not investigate tendons. However, capsaicin induces a pain response when injected to the distal tendon of the tibialis anterior tendon [37].

From the above discussion, it is possible to hypothesise that the increased production of oxidised linoleic acid derivatives may be involved in the pain associated with Achilles tendinopathy as a result of the activation of TRPV1 receptors. This pathway may also be involved in the local effects of the neuropeptide substance P on tendons, given that TRPV1 receptors on sensory nerves gate its release [38,39]. Finally, the oxylipins may produce deleterious changes independently of TRPV1 receptors. 9- and 13-HODE can potentiate epidermal growth factor (EGF)-stimulated DNA synthesis in fibroblast cells [40]. Given that EGF can increase expression of vascular endothelial growth factor in rat embryonic tenocytes, particularly under conditions of hypoxia [41], a study investigating the mitogenic and angiogenic effects of the linoleic acid-derived oxylipins in cultured human tenocytes is clearly warranted.

In conclusion, the present study, albeit exploratory, has identified increased serum levels of linoleic acid-derived oxylipins in Achilles tendinopathy. These findings motivate a more directed study where both the oxylipins and the polyunsaturated fatty acids themselves are investigated in a larger sample of cases with Achilles tendinopathy, and where it may be possible to relate the observed changes to the disease severity, observed histopathology and/or pain scores.

## Supporting Information

S1 Table Oxylipin chemical names and serum levels (in ng/ml) in the present study (“Study”, n = 31) and in two reference studies ([19,20]). (DOCX)

S1 Fig A, Scatterplot and B. ROC plot of the PCA factor derived from a principal component analysis using the mean-centered and Pareto-scaled log_10_ values for 9(S)-HODE, 13-HODE, 9,10-DiHOME, 12,13-DiHOME, 9,10,13-TriHOME and 9,12,13-TriHOME. ***P<0.001, two-tailed t-test. The area under the ROC curve for the PCA factor with respect to the patient category was 0.86 (95% CI 0.73–1, P<0.001).
(TIF)
