# Supplementary material for: Serum Levels of Oxylipins in Achilles Tendinopathy: An Exploratory Study
Source: PLoS One. 2015 Apr 13;10(4):e0123114. doi: 10.1371/journal.pone.0123114 (PMC4395257; doi:10.1371/journal.pone.0123114)
Supplement: S1 Table — (DOCX) [file pone.0123114.s001.docx]

**S1 Table. Oxylipin chemical names and serum levels (in ng/ml) in the present study (“Study”, n=31) and in two reference studies ([19,20]).**

| Abbreviation | Chemical name | Study (median, range) | Ref A (mean±SEM) | Ref B (mean, range) |
| --- | --- | --- | --- | --- |
| *Linoleic acid derivatives* | |  |  |  |
| 9(S)-HODE | 9(S)-hydroxy-10E,12Z-octadecadienoic acid | 2.0 (0.74-13) | 3.4±0.47 | 63 (56-76) |
| 13-HODE | 13-hydroxy-10E,12Z-octadecadienoic acid | 3.5 (1.3-20) | 2.8±0.22 |  |
| 9,10,DiHOME | 9(10)-dihydroxy-12Z-octadecenoic acid | 0.64 (0.22-3.1) | 1.4±0.35 | 2.5 (1.5-3-5) |
| 12,13-DiHOME | 12(13)-dihydroxy-9Z-octadecenoic acid | 1.4 (0.54-7.0) | 0.85±0.08 | 2.5 (1.2-4.1) |
| 9,10,13-TriHOME | 9,12,13-trihydroxy-11-octadecenoic acid | 0.15 (0.047-0.83) | 1.2±0.11 |  |
| 9,12,13-TriHOME | 9,12,13-trihydroxy-10E-octadecenoic acid | 0.53 (0.20-3.4) | 0.99±0.08 |  |
| 9(10)-EpOME | 9(10)epoxy-9Z-octadecenoic acid, leukotoxin | 18 (0-63) |  | 10 (6.2-20) |
| 12(13)-EpOME | 12(13)epoxy-9Z-octadecenoic acid, *iso*-leukotoxin | 12 (4.2-35) |  | 12 (7.1-22) |
| 13-oxo-ODE | 12-oxo-9Z,11E- octadecadienoic acid | 0.61 (0-3.0) | 1.7±0.65 |  |
|  |  |  |  |  |
| *Dihomo-γ-linolenic acid derivative* | |  |  |  |
| 15(S)-HETrE | 15(S)-hydroxy-8Z,11Z,13Z-eicosatrienoic acid | 0.038 (0-0.32) | 0.10±0.03 |  |
|  |  |  |  |  |
| *Arachidonic acid derivatives* | |  |  |  |
| PGD_2_ | 9α, 15(S)-dihydroxy-11-oxo-prosta-5Z,13E-dien-1-oic acid, prostaglandin D_2_ | 0.065 (0-0.43) |  |  |
| PGE_2_ | 9-oxo-11α,15(S)-dihydroxy-prosta-5Z,13E-dien-1-oic acid, prostaglandin E_2_ | 0.14 (0.014-1.8) |  |  |
| PGF_2α_ | 9α,11α,15(S)-trihydroxyprosta-5Z,13E-dien-1-oic acid, prostaglandin F_2α_ | 2.5 (0.14-6.1) |  |  |
| TXB_2_ | 9α,11,15(S)-trihydroxythromba-5Z,13E-dien-1-oic acid, thromboxane B_2_ | 6.5 (0-135) |  |  |
| LTB_4_ | 5(S),12(R)-dihydroxy-6Z,8E,10E,14Z-eicosatetraenoic acid, leukotriene B_4_ | 0.055 (0.008-0.26) |  |  |
| 5-HETE | 5-hydroxy-6E,8Z,11Z,14Z-eicosatetraenoic acid | 0.34 (0.12-1.4) | 0.11±0.038 | 14 (12-16) |
| 8-HETE | 8-hydroxy-5Z,9E,11Z,14Z-eicosatetraenoic acid | 8.1 (0-52) | 0.058±0.032 | 10 (8.7-12) |
| 9-HETE | 9-​hydroxy-​5Z,​7E,​11Z,​14Z-​eicosatetraenoic acid | 0 (0-0.18) | 0.087±0.087 | 18 (15-22) |
| 11-HETE | 11-hydroxy-5Z,8Z,12E,14Z-eicosatetraenoic acid | 0.20 (0.017-2.6) | 0.22±0.13 | 14 (11-18) |
| 12-HETE | 12-hydroxy-5Z,8Z,10E,14Z-eicosatetraenoic acid | 8.9 (0.058-150) | 0.25±0.11 | 15 (12-18) |
| 15-HETE | 15-hydroxy-5Z,8Z,11Z,13E-eicosatetraenoic acid | 0.49 (0.16-4.3) | 0.22±0.038 | 35 (26-43) |
| 20-HETE | 20-hydroxy-5Z,8Z,11Z,14Z-eicosatetraenoic acid | 0.92 (0-3.5) | 0.039±0.026 |  |
| 5-oxo-ETE | 5-oxo-6E,8Z,11Z,14Z-eicosatetraenoic acid | 0 (0-0.21) |  |  |
| 12-oxo-ETE | 12-oxo-5Z,8Z,10E,14Z-eicosatetraenoic acid | 0.38 (0-1.3) |  |  |
| 15-oxo-ETE | 15-oxo-5Z,8Z,11Z,13E-eicosatetraenoic acid | 0.068 (0-0.28) |  |  |
| 5,6-DHET | 5,6-dihydroxy-8Z,11Z,14Z-eicosatrienoic acid | 0.13 (0.04-0.39) | 0.047±0.011 |  |
| 8,9,DHET | 8,9-dihydroxy-5Z,11Z,14Z-eicosatrienoic acid | 0.064 (0.033-0.15) | 0.081±0.017 | 1.0 (0.78-1.3) |
| 11,12-DHET | 11,12-dihydroxy-5Z,8Z,14Z-eicosatrienoic acid | 0.11 (0.047-0.26) | 0.15±0.015 | 0.34 (0.28-0.44) |
| 14,15-DHET | 14,15-dihydroxy-5Z,8Z,11Z-eicosatrienoic acid | 0.41 (0.16-0.70) | 0.17±0.014 | 0.26 (0.21-0.34) |
| 5(6)-EET | 5(6)-epoxy-8Z,11Z,14Z-eicosatrienoic acid | 0.75 (0.20-2.0) |  |  |
| 8(9)-EET | 8(9)-epoxy-5Z,11Z,14Z-eicosatrienoic acid | 0.34 (0.089-0.79) |  | 4.2 (3.2-6.4) |
| 11(12)-EET | 11(12)-epoxy-5Z,8Z,14Z-eicosatrienoic acid | 0.37 (0.14-0.88) |  | 3.8 (1.9-6.1) |
| 14(15)-EET | 14(15)-epoxy-5Z,8Z,11Z-eicosatrienoic acid | 0.27 (0.054-0.80) |  | 3.5 (2.2-5.4) |
|  |  |  |  |  |
| *Eicosapentaenoic acid derivative* | |  |  |  |
| 12(S)-HEPE | 12(S)-hydroxy-5Z,8Z,10E,14Z,17Z-eicosapentaenoic acid | 0.68 (0.060-5.6) | 0.018±0.018 | 1.1 (0.76-1.3) |
|  |  |  |  |  |
| *Docosahenaenoic acid derivatives* | |  |  |  |
| 17(R)-HDoHE | 17(R)-hydroxy docosahexaenoic Acid | 0 (0-1.0) | 0.31±0.11 |  |
| Resolvin D1 | 7(S),8(R),17(S)-trihydroxy-4Z,9E,11E,13Z,15E,19Z-docosahexaenoic acid | 0 (0-0.10) |  |  |
| Resolvin D2 | 7(S),16(R),17(S)-trihydroxy-4Z,8E,10Z,12E,14E,19Z-docosahexaenoic acid | 0.17 (0-0.47) |  |  |

Ref A and Ref B refer to plasma levels (originally reported in nM) found in the studies of Caliguri et al. ([19] “Ref A”, n=10) and of Schuchardt et al ([20], “Ref B”, n=6), included to illustrate the differences in levels that have been reported in the literature. The values for Ref. A are for the young group of individuals (age range 19-28 y) prior to dietary intervention [19]. The values for Ref. B are for the volunteers (age range 20-50 y) at t=0 before dietary intervention [20].
