# Supplementary figures and images for: Serum Levels of Oxylipins in Achilles Tendinopathy: An Exploratory Study
Source: PLoS One. 2015 Apr 13;10(4):e0123114. doi: 10.1371/journal.pone.0123114 (PMC4395257; doi:10.1371/journal.pone.0123114)

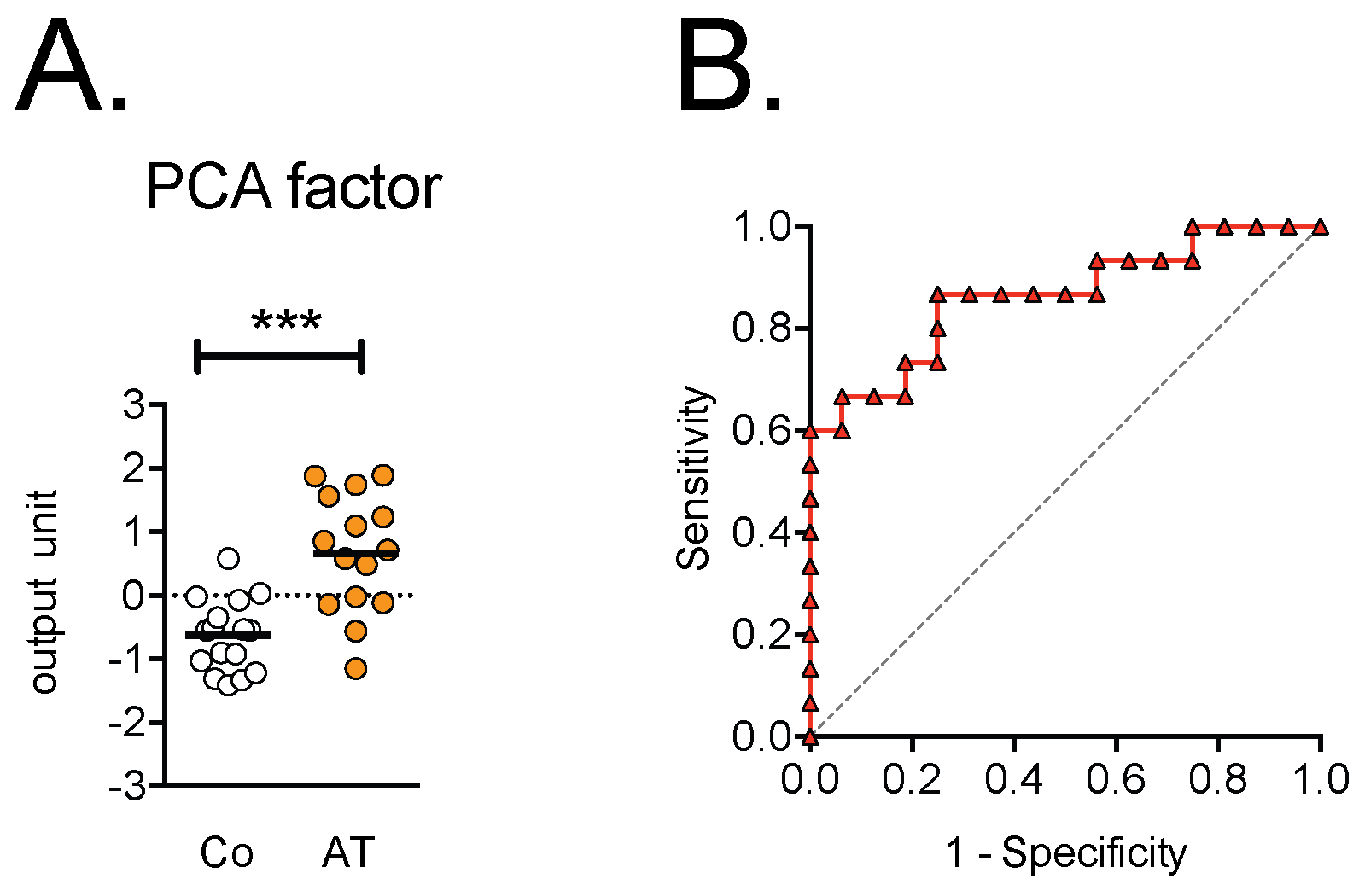

Supplement: S1 Fig — ***P<0.001, two-tailed t-test. The area under the ROC curve for the PCA factor with respect to the patient category was 0.86 (95% CI 0.73–1, P<0.001). (TIF) [file pone.0123114.s002.tif]
